# An iPSC-Derived Myeloid Lineage Model of Herpes Virus Latency and Reactivation

**DOI:** 10.3389/fmicb.2019.02233

**Published:** 2019-10-09

**Authors:** Emma Poole, Christopher J. Z. Huang, Jessica Forbester, Miri Shnayder, Aharon Nachshon, Baraa Kweider, Anna Basaj, Daniel Smith, Sarah Elizabeth Jackson, Bin Liu, Joy Shih, Fedir N. Kiskin, K. Roche, E. Murphy, Mark R. Wills, Nicholas W. Morrell, Gordon Dougan, Noam Stern-Ginossar, Amer A. Rana, John Sinclair

**Affiliations:** ^1^Department of Medicine, University of Cambridge, Cambridge, United Kingdom; ^2^Division of Infection and Immunity, School of Medicine, Cardiff University, Cardiff, United Kingdom; ^3^Department of Molecular Genetics, Weizmann Institute of Science, Rehovot, Israel; ^4^Cleveland Clinic, Lerner Research Institute, Cleveland, OH, United States; ^5^Wellcome Trust Sanger Institute, Hinxton, United Kingdom

**Keywords:** human cytomegalovirus, latency, induced pluripotent stem cells, C2-iPSCs, viral carriage, myeloid, dendritic cells, endothelial progenitor cells

## Abstract

Herpesviruses undergo life-long latent infection which can be life-threatening in the immunocompromised. Models of latency and reactivation of human cytomegalovirus (HCMV) include primary myeloid cells, cells known to be important for HCMV latent carriage and reactivation *in vivo*. However, primary cells are limited in availability, and difficult to culture and to genetically modify; all of which have hampered our ability to fully understand virus/host interactions of this persistent human pathogen. We have now used iPSCs to develop a model cell system to study HCMV latency and reactivation in different cell types after their differentiation down the myeloid lineage. Our results show that iPSCs can effectively mimic HCMV latency/reactivation in primary myeloid cells, allowing molecular interrogations of the viral latent/lytic switch. This model may also be suitable for analysis of other viruses, such as HIV and Zika, which also infect cells of the myeloid lineage.

## Introduction

Herpesviruses are large dsDNA viruses which persist in the infected host for its lifetime. Like all herpesviruses, after primary infection, human cytomegalovirus (HCMV), which is the largest member of the human herpesviruses, is never cleared but persists in its human host with up to 100% of individuals persistently carrying the virus in some populations. As with all herpesviruses, HCMV’s life-long persistence is known to be underpinned by the ability of the virus to undergo a latent infection; this is defined as carriage of the virus in the absence of lytic viral DNA replication or virus production and, in part, helps the virus to persist for the lifetime of the host by escaping immune detection. This ability of HCMV to co-exist with its human host so successfully has also made it a paradigm for analysis of host–pathogen interactions, particularly pathogen immune evasion strategies.

Primary infection or reactivation of HCMV from latency is a major cause of life-threatening disease in virus carriers whose immune systems are compromised or immature (such as HIV/AIDS patients, transplant patients, or the fetus *in utero*) (Sissons et al., 1991). Currently, there is no vaccine against HCMV and no current anti-virals target the latent phase of infection.

*In vivo*, one site of HCMV latent carriage is in CD34+/CD14+ cells of the myeloid lineage. However, differentiation of these cells into dendritic cells (DCs) or macrophages supports reactivation of latent virus. Although primary myeloid cells have been used successfully for the analysis of HCMV latency and reactivation, they can be difficult to obtain and are also difficult to genetically modify which has put substantial limitations on their use. The use of established myeloid cell lines, such as the CD14 + THP1 (Beisser et al., 2001) or CD34 + Kasumi3 (Albright and Kalejta, 2013) cells, has allowed some analysis of HCMV latency and reactivation. However, such cells do not mimic every aspect of HCMV latency when compared to primary cells (Albright and Kalejta, 2013) and these progenitor-like or monocyte-like established cell lines are not fully akin to primary CD34+ or CD14+ cells, and unlike primary cells, are also continuously dividing.

In contrast to primary cells or cell line models, induced pluripotent stem cells (iPSCs) are easily obtained from any individual and capable of differentiation into multiple cell types, including myeloid cells with often identical functions to their primary cell type equivalent (Geti et al., 2012; Ormiston et al., 2015; Sachamitr et al., 2017; Kumar et al., 2018). It has been shown that iPSCs and ES cells are non-permissive for HCMV infection (D’Aiuto et al., 2012; Penkert and Kalejta, 2013; Berger et al., 2015; Cosset et al., 2015; Brown et al., 2019) and that following differentiation of ES or iPSCs to more differentiated cell types prior to infection can result in a permissive infection or that virus can be reactivated from iPSCs by inducing differentiation of the cells into more differentiated cell types (D’Aiuto et al., 2012; Bigley et al., 2013; Nakamura et al., 2013; Penkert and Kalejta, 2013; Belzile et al., 2014; Berger et al., 2015; Cosset et al., 2015; Rolland et al., 2016). Indeed, such models have been used to study HCMV latency and reactivation (Lee et al., 2015, 2016). However, no study has demonstrated that iPSCs can support HCMV latency nor, importantly, model the transition from HCMV latency to reactivation in the disease relevant myeloid lineage. The ability to generate iPSCs from any defined patient genotypes, and their ability to be easily genetically manipulated by technologies such as CRISPR/Cas9, would make an iPSC-derived model of differentiation-dependent HCMV latency and reactivation a powerful tool; allowing a more thorough understanding of how host-specific differentiation-dependent factors control virus latency and reactivation in disease relevant cell types. It could also be a powerful tool to investigate other viruses such as HIV-1 (Maslin et al., 2005) and Zika (Lum et al., 2018) in which the myeloid lineage also plays a major role in virus life cycle.

Here we show that cells derived from a specific iPSC line represent a cell model which can faithfully mimic myeloid differentiation-dependent latency and reactivation of HCMV and can be used to interrogate molecular aspects of HCMV latent carriage and reactivation from latency.

## Results and Discussion

**FIGURE 1:**
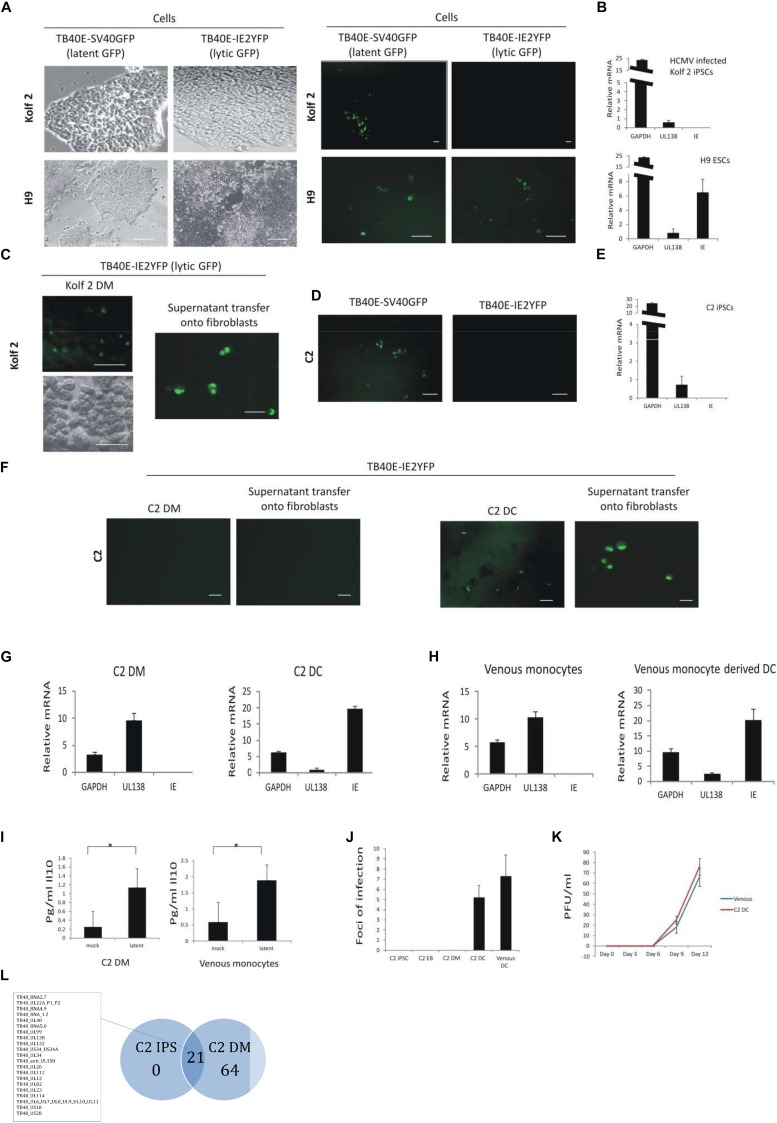
C2 and Kolf2 cells can support a latent infection but only C2 cells maintain latent carriage down the myeloid lineage and can support viral reactivation. Kolf2 and H9 were infected with either TB40E-SV40GFP (MOI 5) (which results in GFP expression in both lytically and latently infected cells) or TB40E-IE2YFP (MOI 5) (which results in GFP expression only in lytically infected cells) and analyzed by fluorescence microscopy at 4 days post infection (brightfield and fluorescence images are shown) **(A)** or RT-qPCR of the SV40GFP (MOI 5) infected cells **(B)**. Additionally, after differentiation to EBs, monocytes derived from Kolf2 cells were infected with TB40E-IE2YFP virus (MOI 5) (**C**, left panel) and supernatants were transferred after 8 days to fibroblasts and analyzed by IF 6 days later (**C**, right panel). C2 iPSCs were also infected with TB40E-SV40GFP (MOI 5) or TB40E-IE2-YFP (MOI 5) and analyzed by fluorescent microscopy **(D)** or RNA qPCR **(E)**. After differentiation to EBs, the derivative C2 iPSC-derived monocytes (C2 DMs) were infected with TB40E-IE2YFP (MOI 5) virus (**F**, left hand panels) and these cells were subsequently differentiated to C2 DCs for 8 days (**F**, right hand panels) and examined for GFP fluorescence directly or co-cultured on indicator fibroblasts as indicated. Alternatively, latently infected C2 DMs were analyzed directly by RT-qPCR (**G**, left panel) or after their differentiation to C2 DCs (**G**, right panel). Latently infected venous monocytes were similarly analyzed directly by RT-qPCR (**H**, left panel) or after their further differentiation to DCs (**H**, right panel). Supernatants from C2 DMs or venous monocytes that had been latently infected with TB40E-SV40GFP (MOI 5) were also analyzed for cellular IL-10 induction by ELISA **(I)**. C2 cells were infected with TB40E-IE2YFP (MOI 5) and then supernatants transferred to fibroblasts at the different stages of myeloid differentiation as indicated. Venous monocytes latently infected with TB40E-IE2YFP (MOI 5) were also differentiated for 8 days to DCs (venous DCs) before transferring supernatants to fibroblasts and foci of infection (indicating reactivation) were enumerated 6 days post-transfer **(J)**. Finally, C2 DMs and venous monocytes (venous) were infected with HCMV (MOI 5) for 4 days before differentiating into DCs and transferring supernatants onto fibroblasts for up to 12 days. Supernatants from these were then used to inoculate fresh fibroblasts to assay virus release **(K)**. Standard error bars are shown and significance determined using Student’s *t*-test where ^∗^*p-*value < 0.05. In photomicrographs, the bar represents 50 μm. Finally, undifferentiated C2 iPSC cells as well as C2 DMs were latently infected with HCMV for 5 days before sorting and analyzing by single cell RNAseq. The average number of reads from 130 latent C2 and 32 latent C2 DMs were averaged and each viral product with a read over 0.05 was enumerated and presented in as a Venn diagram and genes common to latency in both cell types are listed **(L)**. The full dataset is shown in Supplementary Figure 3E. The list of genes common to latency in both cells types are listed.

A single previous analysis has suggested that H9 embryonic stem cells (H9s) can be latently infected with HCMV and reactivated by their differentiation to parietal endoderm by 12-O-tetradecanoylphorbol-13-acetate (TPA) treatment (Penkert and Kalejta, 2013). This model has been used for study of viral UL138 and its role during HCMV latency (Lee et al., 2015, 2016). However, this study did not analyze the carriage of HCMV after differentiation of these cells along the biologically relevant myeloid lineage and still has the inherent disadvantage that, while there are many different ESCs commercially available, using such cells generates political and ethical debate. Additionally, H9 cells cannot be generated from different genotypes in the laboratory. Nevertheless, we used this cell line alongside Kolf2 iPSCs and their myeloid derivatives (Sachamitr et al., 2017; Yeung et al., 2017) as well as C2 iPSCs and their myeloid derivatives (Geti et al., 2012) to compare the suitability of these stem cell lines for studies of HCMV latency and reactivation, particularly in the myeloid lineage. We, firstly, analyzed their side-by-side infection with SV40GFP-tagged HCMV (which is expressed in both latently and lytically infected/reactivated cells (Krishna et al., 2016; Shnayder et al., 2018) and IE2-YFP-tagged HCMV (which is only expressed in lytically infected cells). In our hands, approximately 1% of H9 cells expressed GFP after infection with SV40GFP-tagged HCMV. A similar proportion of cells also expressed IE2YFP after infection with IE2-YFP-tagged HCMV at 4 days post infection, which is indicative of a lytic infection (Figures 1A,B) and inconsistent with the previous report (Penkert and Kalejta, 2013). The reasons for this discrepancy are, at present, unclear to us. One possibility is that these cells had lost their pluripotency which we ruled out as all H9 cells in our study stained positive for Oct4 and Nanog pluripotency markers (Supplementary Figures S1E,F), suggesting this is not due to their partial differentiation. Another possibility is that in the previous report the cells were cultured in different media (Penkert and Kalejta, 2013) which may, perhaps, contain cytokines from conditioned media which the H9 cells are sensitive to. In contrast to H9 cells, Kolf2 iPSCs did not support expression of IE2YFP, suggesting that a latent infection had been established (Figure 1A). In order to confirm that the GFP positive population of cells after infection with SV40GFP-tagged HCMV were truly latent, we also carried out an analysis of viral gene expression by RT-qPCR. This confirmed that infected Kolf2 iPSCs showed detectable levels of expression of the latency-associated gene, UL138, in the relative absence of viral lytic immediate early (IE) gene expression – all indicative of latently infected cells (Figure 1B). In contrast, GFP positive H9 cells, after infection with SV40GFP-tagged HCMV, showed a clear lytic transcription profile with easily detectable levels of IE RNA, akin to a lytic infection, at 4 days post infection (Figure 1B) and also expressed lytic IE2YFP upon infection with IE2-YFP-tagged virus (Figure 1A). On the basis that Kolf2 iPSCs appeared to be latently infected (viral genome positive as assessed by SV40GFP expression but independently negative for IE2YFP expression), we wanted to confirm that infection of the Kolf2 iPSC-derived monocyte-like myeloid cells (Kolf2 DMs), which were cultured identically to the C2-derived myeloid cells (C2 DMs), also resulted in latent infection, as is routinely the case for myeloid precursors *ex vivo* (Sinclair and Poole, 2014). In contrast to peripheral blood monocytes (Krishna et al., 2016) or monocytes derived from primary CD34+ progenitors (Reeves et al., 2005b), Kolf2 DMs, instead, underwent a lytic infection as determined by expression of IE2 after infection with IE2YFP-tagged virus (Figure 1C, left panel) and release of infectious virus which was detectable on indicator fibroblasts when supernatants were transferred 8 days post infection (Figure 1C, right panel). For this reason, Kolf2 iPSC cells do not appear to model myeloid differentiation-dependent HCMV latency and reactivation.

Consequently, we next examined whether C2 iPSCs and their myeloid derivatives (Geti et al., 2012; Kiskin et al., 2018) were a suitable model for latent carriage and reactivation of HCMV. A feeder-dependant version of the C2 iPSC line has been previously published (Kiskin et al., 2018), however, to make our experiments with this line more comparable to the feeder-free H9 and Kolf2 iPSC lines used in this study, we used C2 iPSCs that have been adapted to grow feeder-free and from single cells. As such, it was important to validate that this adapted C2 iPSC line exhibited pluripotency and that it could be differentiated into myeloid-like cells that are comparable to primary venous monocytes and DCs. We found that C2 iPSCs could differentiate into all three germ layers (Supplementary Figures S1A–D) expressed the pluripotency markers OCT4, NANOG, TRA-1-60, and TRA-1-81 (Supplementary Figure S1D). We also found that C2 iPSCs, as well as Kolf2 iPSCs, were able to differentiate into monocyte myeloid cells (C2 DMs) and then, following cytokine-mediated induction, expressed low levels of the DC-associated markers tested (with the possible exception of HLA-DR which had higher levels of expression, as observed with DCs derived from venous monocytes) (Supplementary Figures S2A–C). Therefore, while these cells were not equivalent to venous DCs in terms of marker expression, they did resemble venous DCs and were termed “C2 DCs” here and throughout the manuscript (Supplementary Figures S2A,B).

Furthermore, analysis of cells based on single cell RNAseq using *t*-distributed stochastic neighbor embedding (t-SNE) showed that C2 DMs clustered with venous monocytes as a population quite distinct from their C2 iPSCs parent cells. This confirmed that C2 iPSCs differentiate into cells that are transcriptionally similar to venous monocytes (Supplementary Figure S2D).

Following infection of C2 iPSCs with IE2-YFP-tagged HCMV, no lytic IE2 antigen was detected even though these cells were GFP positive when infected with SV40GFP-tagged virus, consistent with a latent phenotype which was maintained while the cells were cultured as iPSCs in monolayers (Figure 1D). RNA analysis of these latently infected C2 iPSCs also confirmed their latent infection with detectable expression of the latency associated UL138 viral gene in the relative absence of IE gene expression (Figure 1E). Furthermore, infected C2 DMs were also latently infected and only produced virus after their differentiation into C2 DCs as determined by co-culture on fully permissive indicator fibroblasts (Figure 1F). Similarly, RNA analysis of TB40E-SV40GFP infected C2 DMs and C2 DCs also confirmed a latent transcription or lytic transcription profile, respectively (Figure 1G), and this fully mimicked the latent and lytic transcription profile routinely observed in venous monocytes (Reeves et al., 2005a; Figure 1H) or the DCs derived from them, respectively (Figure 1H). Therefore, while the C2 DM cell surface marker expression levels differed slightly from that of venous monocytes, these cells were phenotypically and functionally a suitable model for the support of HCMV lytic infection.

We, additionally, confirmed latent infection of these C2 DMs cells by using a novel red/green, recombinant dual-tagged virus expressing mCherry from the SV40 promoter (which would mark both latently and lytically infected cells) and GFP from an IE2-2A-GFP fusion protein (which will only mark lytically infected cells). When either C2 iPSCs or venous monocytes were infected with this virus, only red cells were detectable, consistent with a latent infection. However, if the cells were differentiated using PMA prior to infection, cells appeared both red and green (Supplementary Figure S2E), consistent with a lytic phenotype. Taken together, these analyses confirmed that C2 DMs support a true latent infection since, in addition to expressing the latent transcript UL138 in the absence of the lytic IE transcript, they also showed no infectious virion production.

To further characterize the latent infection of these cells, both venous monocytes and C2 DMs were tested for their ability to induce cellular IL10 (cIL10) upon infection, which is routinely observed upon latent infection of myeloid cells with HCMV (Avdic et al., 2016). Figure 1I shows that both venous monocytes and C2 DMs induce cIL10 upon HCMV infection at 4 days post infection. Finally, when TB40E-SV40GFP latently infected C2 DMs were differentiated to C2 DCs, they also showed a similar reactivation phenotype to latently infected venous monocytes differentiated to DCs as determined by foci of infection following 8 days of differentiation (Figure 1J) and also showed similar levels of virus release to reactivated venous monocyte derived DCs after supernatants transfer onto indicator fibroblasts (Figure 1K). A hint as to why C2 DMs and Kolf2 DMs show such a different phenotype with respect to their permissiveness for HCMV lytic infection comes from an analysis of their differentiation markers (Supplementary Figures S2B,C). Both C2 and Kolf2 DMs showed the characteristic increase in levels of expression of HLA-DR following terminal differentiation, however, they were not consistent in their levels of expression of other differentiation markers. Previous analyses of myeloid cells differentiated from fibroblast-derived iPSCs (Sachamitr et al., 2017) showed that Kolf2 DMs expressed high levels of CD209 (Sachamitr et al., 2017), consistent with an immature DC phenotype (Neumann et al., 2008) and this was also observed in this analysis (Supplementary Figure S2C). In contrast, C2 DMs expressed CD14 but without CD209, reminiscent of markers observed on intermediate monocytes (Hijdra et al., 2013; Wildgruber et al., 2016; Boyette et al., 2017; Supplementary Figures S2A,B). We think it likely that this difference in permissiveness for HCMV lytic infection between C2 DMs and Kolf2 DMs is due to the less differentiated phenotype of C2 DMs (akin to an intermediate monocyte) compared to the more differentiated immature DC-like phenotype of Kolf2 DMs, especially as immature DCs isolated directly from peripheral blood are known to be permissive for HCMV lytic IE expression after infection *ex vivo* (Hertel et al., 2003). It is also possible that there is some form of differential epigenetic memory in fibroblast-derived iPSCs compared to endothelial-derived iPSCs due to the very nature of their origin (Kim et al., 2010).

Given that different cell types within the myeloid lineage can maintain a latent HCMV infection, we further interrogated the C2 iPSC model to compare the state of latent infection in C2 cells at different stages down the myeloid lineage. To do this, we sorted on the basis of GFP expression for undifferentiated C2 iPSCs and C2 DMs which had been latently infected with TB40E-SV40GFP virus for 5 days. These cells were then analyzed by single cell RNAseq analysis and their extent of latency-associated viral transcription was compared. Firstly, it was clear that latent viral gene expression was more restricted in C2 iPSCs compared to their latently infected derived monocytes with transcription of some 21 viral genes detectable in latently infected C2 iPSCs (Figure 1L). Twenty-one viral genes were detectable in latently infected C2 iPSCs, and all were shared with latently infected monocytes, which also expressed an additional 64 genes (Figure 1L). These data are consistent with previous reports on venous monocytes (Shnayder et al., 2018) where during latency there appears to be a transcription program akin to a late lytic gene expression profile but with low levels of IE gene expression and no production of infectious virus. Here, we show that the viral genes which were expressed in both C2 iPSCs and their derivative C2 DMs include the same viral gene spectrum as those previously shown to be expressed in venous monocytes (Shnayder et al., 2018).

**FIGURE 2:**
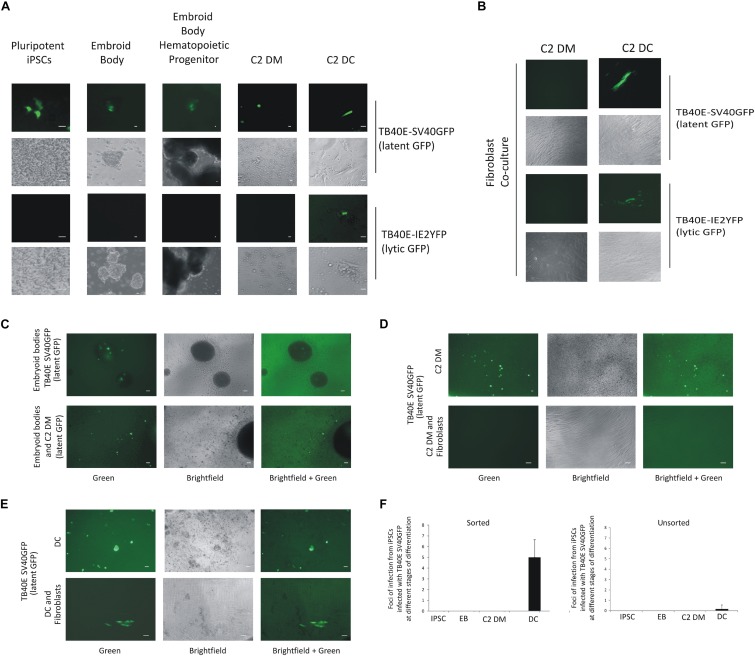
C2 iPSCs infected with HCMV can support HCMV latency and reactivation and can be enriched by FACS sorting of GFP-positive cells. C2 iPSC cells were infected with HCMV viruses TB40E-SV40GFP (MOI 5) (latent GFP) or TB40E-IE2YFP (MOI 5) (lytic GFP) in C2 iPSCs for 4 days in which a latent infection was supported. Cells were then differentiated into embryoid bodies and through to C2 DMs which, after adherence, were further differentiated to C2 DCs, bar represents 5 μm, fluorescence and brightfield images are shown **(A)**. C2 DMs and C2 DCs from these cultures were also assessed for their release of infectious virus by co-culture on indicator fibroblasts, bar represents 5 μm, fluorescence and brightfield images are shown **(B)**. Alternatively, iPSCs infected with TB40E-SV40GFP (MOI 5) (latent GFP) were sorted for GFP and differentiated along the myeloid lineage to EBs and monocytes and assessed for GFP expression, bar represents 50 μm **(C)**. To determine whether these cells were producing infectious virus, the monocytes (C2 DMs) were co-cultured with indicator fibroblasts directly **(D)** or after their further differentiation to DCs **(E)**, bar represents 50 μm. Quantification of release of infectious virus for sorted latently infected C2 iPSCs and their derived EBs, C2 DMs, and C2 DCs are also shown where an average from 10 wells of a 6-well plate were counted and SD shown **(F)**.

On the basis that, upon infection, C2 iPSC cells or C2 DMs both supported a latent infection, we wanted to determine if latently infected C2 iPSCs could maintain this latent infection as they were differentiated firstly to EBs and, then, into monocytes and whether latent virus could subsequently be reactivated from these EB-derived monocytes after their terminal differentiation into DCs. Thus, C2 iPSCs were infected in suspension with SV40GFP-tagged HCMV (to allow visualization of latently infected cells) or IE2-YFP-tagged HCMV (to detect lytically infected cells), before adherence onto plastic in pluripotency media. Following the establishment of latency for 4 days, cells were transferred to low adherence plates to allow EB formation. EBs were, subsequently, differentiated along the myeloid lineage until such time as myeloid precursor cells expressing monocyte markers (Supplementary Figures S2A,B) were released from the EBs as suspension cells (Silk et al., 2011); in contrast, macrophages adhered to the plastic (Supplementary Figure S2; Silk et al., 2011). The C2 DMs were then separated from the EBs by filtration and seeded onto plastic as described previously (Silk et al., 2011). It was possible, at this point, to detect SV40GFP-tagged virus in monocytes but only at extremely low frequency (∼1 in 1 × 10^6^). Nevertheless, consistent with latent carriage of virus in these cells, C2 iPSCs infected with IE2-YFP-tagged virus showed no IE2-YFP expression neither did EBs nor monocytes derived from them (representative fields of view are shown in Figure 2A).

While infected C2 iPSCs and their derivative C2 DMs appeared to be carrying latent virus, a critical test was to confirm that these cells were not producing infectious virus but, importantly, that infectious virus could be reactivated from them after differentiation to C2 DCs. Consequently, we also co-cultured C2 DMs and their C2 DC derivatives generated from EBs derived from infected C2 iPSCs on indicator fibroblasts. Figure 2B clearly showed that, while C2 DMs showed no evidence of release of infectious virus, their differentiation to C2 DCs resulted in clear infectious virus reactivation. Finally, as a further control for the SV40 promoter as an indicator of latency, the C2 DMs were also infected with a TB40E virus which expresses mCherry from the GATA2 promoter (a known myeloid transcription factor). No virions were produced, as assayed by co-cultured on fibroblasts, from C2 DMs infected with any of the viruses unless the C2 DMs were subsequently terminally differentiated into C2 DCs (Supplementary Figures S3A,B). It is worth noting that levels of GATA2, itself, were quite similar in venous monocytes and in C2 DMs (Supplementary Figure S3C) and, as expected mCherry expression, driven by GATA2 in the GATA2-mCherry virus infected C2 DMs was clearly detectable. Similarly, GFP was found to be expressed in C2 DMs cells infected with SV40GFP virus whereas C2 DMs infected with TB40E-IE2YFP showed no IE2YFP expression. Additionally, full RNAseq analysis of viral transcripts showed that the same range of latency associated transcripts was found as previously observed for venous monocytes (Shnayder et al., 2018; Supplementary Figures 3D,E and Supplementary Methods).

Taken together, these analyses showed that infected C2 iPSCs are able to carry latent virus through EB differentiation to monocytes, which can then be reactivated upon terminal differentiation of these monocytes to DCs. Although this culture system provided a reliable model to interrogate the processes of virally latency and reactivation, the frequency of latently infected monocytes released from EBs (Figure 2A) and recovery of infectious virus from reactivating DCs (Figure 2B) were both severely limited by the low initial proportion of latently infected C2 iPSCs (∼1%). Consequently, we modified the protocol to include GFP sorting of the C2 iPSCs latently infected with SV40GFP-tagged virus prior to EB formation to enrich for latently infected cells. Figure 2C shows that this resulted in easily discernible GFP positive cells within the EBs, which were then released at much higher frequency into the monocyte population. To confirm that no infectious virus was being produced from these likely latently infected monocytes, and that they could be differentiated to DCs to induce reactivation, we co-cultured these C2 DMs and their derived C2 DCs on indicator fibroblasts. Figure 2D shows that C2 DMs generated from GFP sorted C2 iPSCs infected with SV40GFP-tagged virus were indeed latent and did not produce infectious virus. In contrast, C2 DCs derived from these C2 DMs after differentiation resulted in clearly discernible reactivation of infectious virions (Figures 2E,F). Thus, GFP-sorted C2 iPSCs carry latent HCMV and are able to form EBs to give rise to C2 DMs which maintain this latent infection but which are then able to reactivate infectious virus production after their terminal differentiation to C2 DCs at approximately 100-fold higher rates than unsorted cells (Figure 2F). This makes them particularly suitable for following latency and reactivation along the myeloid lineage, which is also known to occur *in vivo* (Hahn et al., 1998; Reeves et al., 2005b; Sinclair and Poole, 2014).

**FIGURE 3:**
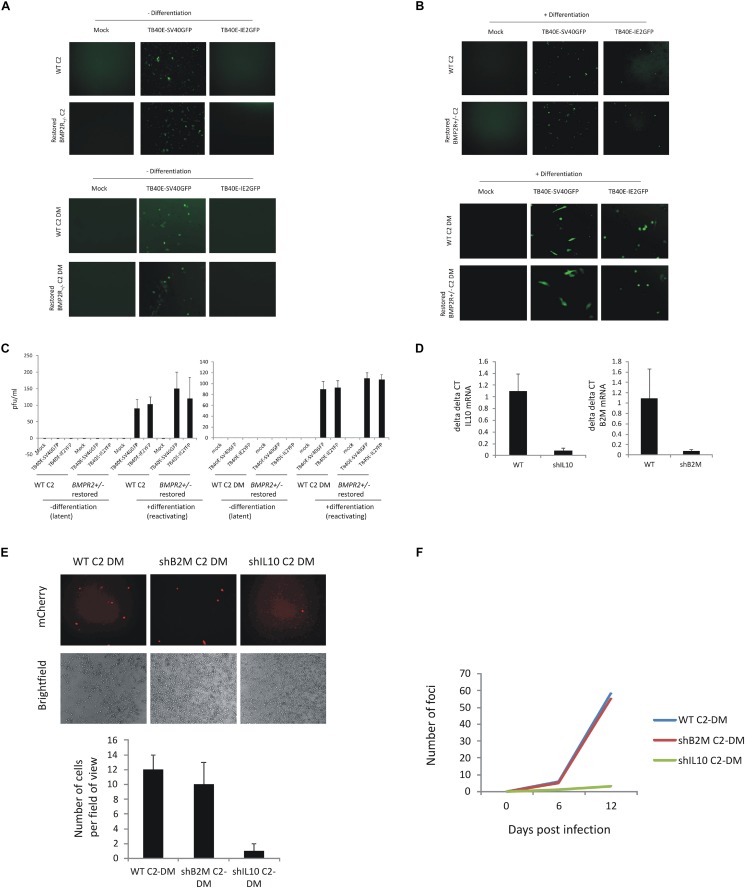
C2 iPSCs retain the ability to support HCMV latency and reactivate virus after CRISPR/Cas9 editing and removal of cellular IL10 by lentiviral shRNA decreases HCMV maintenance and reactivation. C2 iPSCs or C2 iPSCs which have been mutated using CRISP-R directed gene editing (*BMPR2* ± C2 iPSCs or derived monocytes) were restored for WT BMP2 levels using 50 ng/ml BMP4 (BioTechne) before infecting with either TB40E-SV40GFP (MOI 5) or TB40E-IE2YFP (MOI 5) and during which time latency is supported for 4 days **(A)** before differentiating to induce reactivation **(B)**. Supernatants were collected and virion production was assessed by transferring to fibroblasts **(C)**. C2 iPSCs were also lentivirally transduced with lentiviral vectors delivering shIL10 or shB2M to cells. Following establishment of the C2 shIL10 and B2M knockout cell line, cells were differentiated into C2 DMs and validated by RTqPCR alongside WT C2 DMs **(D)**. WT C2 DM, B2M C2 DM, and shIL10 C2 DM cells were then latently infected with TB40E-GATA2-mCherry strain of HCMV (MOI 5) and analyzed by immunofluorescence 6 days later for the presence of the mCherry marker and fields of view were enumerated **(E)**. The release of virus was assessed following differentiation of the C2 DMs into C2 DCs and analyzing the formation of foci of infection with fibroblasts **(F)**.

Our view is that these C2 iPSCs, which could be easily manipulated by current gene editing technologies (Gonzalez, 2016; Kiskin et al., 2018), will be an invaluable resource to study cellular and viral factors required for establishment and maintenance of latent HCMV infection as well as virus reactivation. Indeed, CRISPR/Cas9 editing of iPSCs has already been successfully used to study HIV-1 in macrophages (Taylor et al., 2018). While the process of CRISPR/Cas9 editing should not in itself affect the ability of cells to support latency, to control for any such non-specific effect, C2 iPSC cells which had been CRISPR/Cas9 edited (Kiskin et al., 2018) were tested for the ability to support latency and to reactivate. Figure 3 shows that CRISPR-/Cas9-edited C2 iPSCs do indeed retain the ability to support latency (based on the absence of GFP+ cells after infection with TB40E-IE2GFP, Figure 3A) and reactivate infectious virus when differentiated using PMA, a known inducer of HCMV reactivation in many cell types (Delannoy et al., 1997; Penkert and Kalejta, 2013) (based on the presence of GFP+ cells after infection with TB40E-IE2GFP, Figure 3B). Again, the ability of these cells to support latency and reactivation was validated by their inability or ability to produce infectious virions (Figure 3C, infectious virions are produced from iPSCs treated with PMA when infected with TB40E-SV40GFP or TB40E-IE2GFP). Furthermore, Figures 3A–C also show that C2 DM cells differentiated from latently infected, CRISPR-edited iPSCs, can support latency and reactivation. Thus, these iPSCs can be successfully manipulated using CRISPR without losing the ability to support HCMV latency and reactivate virus after their differentiation to DC-like cells.

As another proof of principle, we also used shRNA lentiviral transduction, which has been successfully used previously to knock down genes in iPSCs (Kamata et al., 2010) to target the cIL10 gene on the basis that cIL10 expression has previously been shown to be important for maintenance of latent viral genome carriage in primary myeloid cells (Mason et al., 2012; Poole et al., 2014a; Avdic et al., 2016). Figure 3D shows that lentiviral delivery of shRNAs to cIL10 in C2 iPSCs decreased cIL10 expression and this had a negative impact on the maintenance of latent viral genomes (compare left-hand panel to the right-hand panel in Figure 3E) as well as reactivation of infectious virus after differentiation of these latently infected C2 iPSCs (Figure 3F). The effects were not due to shRNA transduction as when the same experiment was carried out with C2 cells that had been transduced with lentiviral particles encoding shRNA to the beta-2 microglobulin (B2M) gene, there was no effect on the ability of the cells to maintain the viral genome (Figures 3D–F). The successful differentiation of these cells along the myeloid lineage also makes them ideal candidates for the study of other viruses, which can also infect myeloid lineage cells, such as HIV-1 and Zika. Furthermore, since these cells are pluripotent, and can potentially differentiate along multiple lineages, they could also be used to address the ongoing question of other potential sites of HCMV latency besides the myeloid lineage (Sinclair and Poole, 2014) or for the study of latency and reactivation of other viruses which are also cell differentiation dependent.

## Materials and Methods

### Cells

The healthy control human iPSC (hiPSC) line Kolf2 (fibroblast derived) was acquired through the human iPSCs Initiative Consortium (HipSci^1^). H9 and Kolf2 cells and differentiation along the myeloid lineage has been described previously (Silk et al., 2011).

In brief, H9 cells (WiCell) were cultured as previously described (Vallier et al., 2004). Cells were cultured on gelatin in KSR medium made with advanced DMEM/F12 (Gibco) and supplemented with 20% knock-out serum replacement (Gibco), L-glutamine 200 nM (Gibco), activin (4 ng/ml), and FGF2 (10 ng/ml). The iPSCs were adapted to feeder-free culture as follows: C2 iPSCs were originally cultured as colonies on MF1 mouse embryonic fibroblasts (MEFs) (250,000 per well of a six-well plate) in KSR [advanced DMEM/F12 (Gibco) and supplemented with 20% knock-out serum replacement (Gibco), L-glutamine 200 nM (Gibco), activin (4 ng/ml), and FGF2 (10 ng/ml)]. Colonies were detached using collagenase IV + dispase II enzyme mix, which was washed out using serial dilution, and then passaged by trituration. For Week 1, iPSCs were transferred onto 187,500 MEFs per well of a six-well plate, and fed with 75% KSR medium + 25% MEF-conditioned chemically defined medium (CDM)-bovine serum albumin (BSA) medium + 12.5 ng/ml FGF2 + 10 ng/ml activin A + 50 μg/ml ascorbic acid. After 1 week, colonies were passaged using collagenase IV + dispase II enzyme mix and trituration. For Week 2, hiPSCs were transferred onto 125,000 MEFs per well of a six-well plate, and fed with 50% KSR medium + 50% MEF-conditioned CDM-BSA medium + 15 ng/ml FGF2 + 10 ng/ml activin A + 50 μg/ml ascorbic acid. After 1 week, colonies were passaged using collagenase IV + dispase II enzyme mix and trituration. For Week 3, hiPSCs were transferred onto 62,000 MEFs per well of a six-well plate, and fed with 50% KSR medium + 25% MEF-conditioned CDM-BSA medium + 25% CDM-BSA medium + 20 ng/ml FGF2 + 10 ng/ml activin A + 50 μg/ml ascorbic acid. After 1 week, colonies were passaged using collagenase IV + dispase II enzyme mix and trituration. For Week 3, hiPSCs were transferred onto gelatin-coated plates with no MEF feeder cells, and fed with 50% KSR medium + 50% CDM-BSA medium + 20 ng/ml FGF2 + 10 ng/ml activin A + 50 μg/ml ascorbic acid (“BK medium”). After 1 week, colonies were passaged using collagenase IV + dispase II enzyme mix, and then made into single cells using TrypLE Select + 2 mM EDTA. Cells were laid down as single cells into gelatin-coated plates with no MEF feeder cells, and then maintained as such [passaging using TrypLE Select + 2 mM EDTA, and fed using 50% KSR medium + 50% CDM-BSA medium + 20 ng/ml FGF2 + 10 ng/ml activin A (“BK” medium)] for use in experiments. CDM was prepared as previously described (Johansson and Wiles, 1995) and supplemented with BSA. Briefly, CDM-BSA was made by making a 1:1 ratio of IMDM:Ham’s F-12 Nutrient Mixture and adding GlutaMAX-I (Gibco), 0.5% BSA, 1% chemically defined lipid concentrate (100×) (Gibco), 1% antibiotic-antimycotic (100×), Gibco 15240-062, Transferrin (15 μg/ml, Sigma), rh-insulin (7 μg/ml, Sigma), and 1-thioglycerol, 450 μM final concentration (Sigma). C2 iPSC feeder-free medium comprised 1:1 ratio of CDM-BSA:KSR (without activin and FGF2), supplemented with 12.5 ng/ml activin A, 15 μl/ml FGF2, and Vitamin C.

Kolf2 or C2 iPSCs, grown feeder free, were removed from the plastic using ReLeSR medium (Stem Cell Technologies) (C2 cells could also be removed from plastic using trypsin) in the presence of Rock Inhibitor (Stem Cell Technologies). Cells were seeded at 3 × 10^6^ cells per well of a six-well plate in iPSC 4 ml media supplemented with 50 ng/ml bone morphogenic protein 4 (BMP4) (BioTechne), 50 ng/ml VEGF (Peprotech), 20 ng/ml SCF (Bio Techne), and 50 ng/ml GM-CSF (Peprotech). On day 2, 2 ml X-Vivo-15 medium (Lonza) supplemented with 1 mM sodium pyruvate (Life Technologies), 0.1 mM MEM non-essential amino acids (Gibco), 2 mM L-glutamine, 5 μM 2-mercaptoethanol, and growth factors BMP4, VEGF, SCF, and GM-CSF was added to each well. Then 2 ml of media was replaced each 2–3 days using X-Vivo-15 supplemented with VEGF, SCF, and GM-CSF until day 12. After day 12, media was replaced every 2–3 days using X-vivo-15 supplemented with SCF and GM-CSF. On day 19 2 ml of media was replaced with X-vivo-15 supplemented with GM-CSF only. On day 23 the media plus embryoid bodies were filtered with a 70 μM strainer to allow isolation of the single cells (monocytes). The monocytes were then washed in PBS (without magnesium or calcium) and then plated at 1.5 × 10^6^ per well of a six-well cell bind plate in a volume of 4 ml of X-Vivo-15. These cells were then ready for virus infection or terminal differentiation into DCs.

Isolation of primary monocytes from venous blood has also been described (Mason et al., 2012; Sachamitr et al., 2017).

Late outgrowth endothelial progenitor cells (L-EPCs) were de-differentiated to C2 cells as described previously (Geti et al., 2012; Ormiston et al., 2015). C2 cell re-differentiation to embryoid bodies and monocytes were essentially as described for Kolf2 cells (Silk et al., 2011; Sachamitr et al., 2017).

Co-culture of cells on indicator fibroblasts to assess virus production has been described previously (Poole et al., 2014b).

### Viruses

TB40E-SV40GFP (expressing GFP under the control of the SV40 promoter and therefore detectable during latent infection) has the GFP gene inserted between the US34 and TRS1 genes of TB40E. TB40E-IE2YFP (expressing the lytic gene, IE, fused to YFP and detectable during a lytic infection) and TB40E-SV40mCherry has been described previously (Straschewski et al., 2010; O’Connor and Murphy, 2012; Krishna et al., 2017). Dual-tagged virus expressing IE2-GFP separated by the T2A self-cleaving peptide with an mCherry driven constitutive reporter has been published previously (Roche et al., 2018). In this virus the GFP is not fused to IE2 but instead is cleaved due to a 2A protease site between the GFP and the IE2 genes. All virus infections were carried out at an approximate MOI of 5 unless otherwise stated.

### Virus Infection

Cell types were all exposed to an approximate MOI of 5 for 1 h before removing virus. Undifferentiated ECs, iPSCs, or CD34+ cells were infected in suspension at 37 degrees, while adherent cells were infected at RT for 1 h on a rocker. The inoculum was then removed and the cells washed three times in media before allowing latency to leaving the cells for 4 days, during which time a latent infection is supported. For differentiation experiments in iPSCs, monolayers of iPSCs were infected with HCMV, which support a latent infection, in the presence of pluripotency media for 4 days. Cells were then either trypsinised before forming EBs directly or trypsinised and sorted for GFP into media before the formation of EBs.

### CRISPR-Cas9 Targeting in C2 iPSCs

Feeder-free C2 iPSC were targeted using CRISPR-Cas9 as previously described in Kiskin et al. (2018). Briefly, C2 iPSCs previously adapted to grow feeder-free were electroporated with CRISPR-Cas9 plasmids using the Amaxa Nucelofector system. Correctly targeted cells were selected for by neomycin selection and expanded in feeder-free conditions as described in other parts of the section “Materials and Methods.”

### shRNA Targeting in C2 iPSC Cells

Lentiviral vectors encoding shRNAs to B2M and IL-10 were obtained from Santa Cruz. The shRNAs were then transduced into C2 iPSCs. Stable cells lines were generated according to the manufacturer’s protocol (Santa Cruz) except that the media was changed every 24 h (rather than every few days) because C2 iPSCs require feeding every 24 h.

### ELISA

Human IL10 high sensitivity ELISA was used following the manufacturers protocol (Life Technologies).

### RT-qPCR

RT-qPCR analyses for viral gene expression have been described previously (Lau et al., 2016).

## Data Availability Statement

All datasets analyzed for this study are included in the manuscript/Supplementary Files.

## Ethics Statement

All human samples were obtained under ethical approval and after approval of protocols from the Cambridgeshire 2 Research Ethics Committee (REC reference 97/092) conducted in accordance with the Declaration of Helsinki. Informed written consent was obtained from all of the volunteers included in this study before providing blood samples and all experiments were carried out in accordance with the approved guidelines.

## Author Contributions

BL, EM, KR, MW, SJ, and JSh performed the experiments. EP, CH, AB, JF, MS, AN, DS, NS-G, FK, NM, and BK performed the experiments and analyzed the data. EP, JSi, AR, and GD designed the experiments and analyzed the data. EP, AR, and JSi wrote the manuscript.

## Conflict of Interest

The authors declare that the research was conducted in the absence of any commercial or financial relationships that could be construed as a potential conflict of interest.

## Footnotes

1 www.hipsci.org
