# Supplementary material for: An iPSC-Derived Myeloid Lineage Model of Herpes Virus Latency and Reactivation
Source: Front Microbiol. 2019 Oct 9;10:2233. doi: 10.3389/fmicb.2019.02233 (PMC6795026; doi:10.3389/fmicb.2019.02233)
Supplement: Supplementary file 2 [file Data_Sheet_1.docx]

**Supplementary figure legends**

Supplementary Figure 1

**C2 iPSCs express pluripotency markers and can be differentiated into all three germ layers.**

C2 iPSCs can undergo tri-lineage germ layer differentiation expressing Sox17, Brachyury, and Otx2 when differentiated along the endoderm, mesoderm and ectoderm pathways respectively, or none of these when undifferentiated (1A-D). C2 iPSCs express pluripotency markers NANOG, OCT4, TRA-1-60 and TRA-1-80 and H9 cells express pluripotency markers NANOG and OCT4(1E and 1F) White bar represents 50µm.

Supplementary Figure 2

**C2 iPSCs can be differentiated along the myeloid lineage via EBs into C2 DMs and C2 DCs and support an HCMV latent phenotype prior to differentiation.** Differentiation of C2s to EBs, C2 DMs and C2 DCs was assessed morphologically by microscopy, line represents 50µm (2A) and cell surface markers of C2 DMs and C2 DCs from these cultures were compared to markers on venous blood monocytes and their derivative DCs by FACS. The specific FACS plots for 2B are also shown where unfilled represent isotype controls, grey represents myeloid precursors and red represents terminally differentiated cells (2B). Kolf2 cells were also differentiated into Kolf2 DMs (Kolf2 DM) and Kolf2 DCs (Kolf2 DC) and phenotyped by FACS with staining for CD14, CD209 and HLA-DR or unstained, as labelled (2C). Additionally, t-SNE plot based on single cell RNAseq of C2 iPSCs (iPSC, green), venous monocytes (VM, blue) and C2 DMs (DM, red) is shown (2D). Finally, iPSCs or venous monocytes were infected with dual tagged TB40E SV40-mCherry/ IE2-2A-GFP virus before or after their differentiation with PMA and analysed for red and green fluorescence, as indicated, 4 days post infection. Overlays are shown where dual fluorescence was observed (2E).

Supplementary Figure 3

**A recombinant virus expressing mCherry under control of a GATA2 responsive promoter expresses mCherry as efficiently as GFP from an SV40GFP virus.** C2 iPSCs were differentiated into C2 DMs before infecting with either TB40E-GATA2mCherry (MOI 5), TB40E-IE2YFP (MOI 5) or TB40E-SV40GFP (MOI 5). Fluorescent marker expression was analysed after 4 days of latency (left hand panels, 3A), following differentiation into C2 DCs (middle panels, 3A) and virion production was determined by transferring supernatants onto indicator fibroblasts (right hand panels, 3A and 3B). Additionally, levels of GATA2 in both venous monocytes as well as C2 DMs were analysed by western blot (3C). Finally, RNAseq data from infected C2 DMs were analysed for levels of viral gene expression during latency. Graph shows average readings from 32 latent C2 DM cells (3D). Single cell RNAseq analysis of all viral transcripts in these cells are shown in supplementary table 3E.

**Supplementary methods**

*FACS*

FACS analyses have been described previously [41].

*Three germ layer differentiation*

Differentiation of iPSCs to mesoderm (brachyury), endoderm (Sox17) and ectoderm (Otx2) cells was carried out using the R&D systems kit using the manufacturer's protocol and antibodies. Antibodies for Nanog, Oct4, TRA-1-60 and TRA-1-81 were obtained from Stemgent with Alexa 488 secondary antibobies from Lifesciences.

*BMPR2+/- CRISP-R edited cell line*

Wild Type (WT) C2 iPSCs were mutated using CRISP-R directed gene editing the C2 cells used in this manuscript (*BMPR2+/-* C2 iPSCs) by introducing a premature stop codon into one allele of the *BMPR2* gene. Normal levels of BMPR2 can be restored in this mutant line by treating with BMP4 to restore normal levels of BMPR2 in the mutant line. These cells were then infected with either TB40E-SV40GFP or TB40E-IE2YFP and leaving the cells for 4 days, during which time a latent infection is supported, before treating with PMA to induce reactivation. Supernatants were collected and virion production was assessed by transferring to fibroblasts.

*cIL10 and B2M shRNA knock down cell lines*

Lentiviral particles were obtained from Santa Cruz and transduced into C2 cells using the manufacturer's protocol and selecting in 2ug/ml puromycin for 10 days, changing media and antibiotics every 24h. Cells were validated using RTqPCR.

*RNAseq* RNAseq analysis was carried out as described previously [22]. Briefly, single-cell sorting and RNA libraries preparation were done with the MARS-seq protocol using 3'-PolyA primers, essentially as described [22] Briefly, cells from HCMV infected populations of C2 iPSCs, C2 DMs and venous monocytes were FACS sorted into individual wells of 384-well capture plates, barcoded, converted into cDNA and pooled using an automated pipeline. The pooled samples were then linearly amplified by T7 in vitro transcription and the resulting RNA was fragmented and converted into sequencing ready library by tagging the samples with pool barcodes and Illumina sequences during ligation, reverse transcription and PCR. Libraries were tested for quality and concentration as previously described [50] and sequenced on NextSeq 500 (Illumina).

*MARS-seq analysis* The analysis of the MARS-seq data was done as described before in [22]. The reference genome was created from the hg19 and TB40E (NCBI [EF999921.1](https://www.ncbi.nlm.nih.gov/nuccore/EF999921%2e1)). The transcription units of the virus were based on NCBI annotations, with some changes based on the alignment results. The read itself (of length 37bp) was aligned with the reference genome using Bowtie 2, and the counting of the reads per gene was done based on unique molecular identifiers (UMIs) (8 bp). Wells with <1,000 reads were discarded, and genes of ribosomal proteins, histones, and mitochondrial were excluded. Cells analysed for latency had less than 0.5% viral reads.

*t-SNE analysis* The t-SNE plot was calculated with strong enough genes with at least 1,000 UMIs overall cells, after down-sampling each cell to 1,000 UMIs.

Supplementary Figure 1

1F

Supplementary figure 2

2C 2D

2E

Supplementary figure 3

3A

3B

3C

3D

3E (see attached Excel sheet)
